# Differential processing of risk and reward in delinquent and non-delinquent youth

**DOI:** 10.1093/scan/nsad040

**Published:** 2023-08-12

**Authors:** Natasha Duell, Michael T Perino, Ethan M McCormick, Eva H Telzer

**Affiliations:** Department of Psychology and Neuroscience, University of North Carolina at Chapel Hill, Chapel Hill, North Carolina 27514, United States; Frank Porter Graham Child Development Institute, University of North Carolina at Chapel Hill, Chapel Hill, North Carolina 27514, United States; Department of Psychiatry, Washington University School of Medicine in St. Louis, Missouri 63110, United States; Institute of Psychology, Leiden University, Leiden 2333 AK, The Netherlands; Department of Psychology and Neuroscience, University of North Carolina at Chapel Hill, Chapel Hill, North Carolina 27514, United States

**Keywords:** delinquency, adolescents, risk-taking, fMRI, ventral striatum

## Abstract

The present study examined the behavioral and neural differences in risky decision-making between delinquent (*n* = 23) and non-delinquent (*n* = 27) youth ages 13–17 years (*M* = 16, SD = 0.97) in relation to reward processing. While undergoing functional neuroimaging, participants completed an experimental risk task wherein they received feedback about the riskiness of their behavior in the form of facial expressions that morphed from happy to angry. Behavioral results indicated that delinquent youth took fewer risks and earned fewer rewards on the task than non-delinquent youth. Results from whole-brain analyses indicated no group differences in sensitivity to punishments (i.e. angry faces), but instead showed that delinquent youth evinced greater neural tracking of reward outcomes (i.e. cash-ins) in regions including the ventral striatum and inferior frontal gyrus. While behavioral results show that delinquent youth were more risk-averse, the neural results indicated that delinquent youth were also more reward-driven, potentially suggesting a preference for immediate rewards. Results offer important insights into differential decision-making processes between delinquent and non-delinquent youth.

## Introduction

Although some amount of risk-taking during adolescence is normative (Moffitt and Caspi, 2001; Rutter *et al.*, 2006), a small proportion of youth engage in persistent levels of antisocial behavior, hereafter referred to as delinquency (Frick and Viding, 2009; Cook *et al.*, 2015). Delinquent behavior during adolescence is associated with long-term consequences, including poor health, job insecurity and greater risk of lifetime criminality (Bongers *et al.*, 2008; DeLisi *et al.*, 2010; Drury *et al.*, 2019). Despite decades of research examining risk behavior in adolescence, questions remain about what distinguishes youth engaging in normative versus delinquent patterns of risk behavior. Sensitivity to rewards and punishments plays a key role in motivating risk behavior (Bacon *et al.*, 2018). One theory is that delinquent youth are more sensitive to rewards than non-delinquent youth (Byrd *et al*., 2014). This heightened sensitivity to reward may compel delinquent youth to pursue rewarding stimuli with little regard for potential consequences (Frick *et al.*, 2003), or it may curtail effective self-regulation by motivating an impulsive drive for immediate gratification (Steinberg, 2008; Matthys *et al.*, 2013). To unpack how delinquent and non-delinquent youth process rewards during risk-taking, the present study compared behavioral and neural correlates of risky decision-making.

Risk-taking is consistently associated with heightened reward sensitivity (for a review, see Shulman *et al*., 2016). During adolescence, subcortical brain regions responsible for processing rewards, such as the ventral striatum (VS), undergo significant changes that render the adolescent brain highly sensitive to rewards (Schreuders *et al.*, 2018). Experimental studies with adolescents have found evidence of heightened activation in reward-sensitive brain regions when adolescents take risks (Van Leijenhorst *et al.*, 2010) and when they receive rewards (Schreuders *et al.*, 2018). This link between reward-related brain activation and risk-taking has been observed among both delinquent (Bjork *et al.*, 2010) and non-delinquent (Barkley-Levenson and Galvan, 2014) youth. However, some research suggests that delinquent youth may be disproportionately sensitive to rewards (Hawes *et al.*, 2021). For example, on experimental gambling tasks, delinquent youth favor gambles with high rewards even though these gambles ultimately lead to large losses (Lane and Cherek, 2001). Furthermore, upon receiving rewards for their decisions, delinquent youth evince greater VS activation than non-delinquent youth (Bjork *et al.*, 2010). Thus, results from studies using experimental tasks and fMRI suggest that delinquent youth demonstrate more exaggerated behavioral and neural sensitivity to rewards than non-delinquent youth.

Exaggerated reward sensitivity among delinquent youth could lead to increased risky behavior through several pathways. Perhaps increased reward responsivity drives increased thrill-seeking behavior. For example, heightened reward sensitivity is associated with an increased propensity to seek out novel and thrilling experiences (Roose *et al.*, 2011). Prior work has shown that delinquent youth demonstrate a higher propensity for thrill-seeking behaviors than their non-delinquent peers (Castellanos-Ryan *et al.*, 2011). From this perspective, thrill-seeking may dampen sensitivity to the potential consequences associated with risky decision-making (Frick *et al.*, 2003). In line with this, the response modulation hypothesis (Gorenstein and Newman, 1980) postulates that delinquent youth possess a dominant, reward-oriented response set, such that rewarded experiences are more salient than punishments, and therefore do not produce avoidance behavior. This is supported by empirical work showing that delinquent youth show performance deficits in the face of competing reward and punishment (Byrd *et al.*, 2014). For example, on an experimental gambling task, boys with oppositional defiant disorder were more likely than their peers to continue choosing risky options, even after those choices yielded more punishments than rewards (Matthys *et al*., 2004).

Alternatively, heightened reward sensitivity in delinquent adolescents may be associated with a preference for immediate gratification. This preference for immediate rewards may be heightened among delinquent youth due to impairments in the ability to delay gratification (Piquero *et al.*, 2018), which may reflect a strategy of delinquent youth pursuing smaller, more immediate rewards, even if larger rewards could be possible with more effort or time. Decisions involving immediately available rewards are associated with heightened activation in subcortical brain regions implicated in reward processing, including the VS (McClure *et al.*, 2004). Neural systems responsible for processing and responding to rewards may interact with motor systems to increase the likelihood of quick, rather than thoughtful and calculated, responses (Luna, 2012). From this vantage point, risky, reward-oriented decision-making among delinquent youth may be more about the desire to pursue immediate rewards than about insensitivity to punishments.

Examining the neural mechanisms associated with risk-taking in the context of reward may shed light on the psychological processes that drive behavioral differences between delinquent and non-delinquent youth. To this end, the present study had two key aims: (i) to identify behavioral differences in risk-taking between delinquent and non-delinquent youth and (ii) to identify differences in neural sensitivity when taking risks and receiving rewards. To answer these questions, delinquent and non-delinquent youth completed the Social Analog Risk Task (SART), which is modified from the well-validated Balloon Analog Risk Task (BART; Lejuez *et al.*, 2002). During the task, participants took greater risks to obtain larger rewards. Each reward brought the participant closer to the end of the trial. If the trial ended before the participant decided to cash in their earnings, they lost all rewards for that trial. Participants received dynamic feedback on the amount of risk they incurred via facial expressions that morphed from happy (no risk) to angry (high risk), where higher risk indicated being closer to the end of the trial. Thus, the task allowed us to examine neural sensitivity both when making risky decisions and when cashing out to obtain rewards. Behaviorally, we anticipated that delinquent youth would take more risks than non-delinquent youth because they were more sensitive to rewards than the amount of risk they incurred (as indicated by facial expressions). At the neural level, we explored whether delinquent youth demonstrated differential neural sensitivity to increasing risks and increasing rewards relative to non-delinquent youth. Examining the neural correlates of risky decision-making will help clarify the neuropsychological processes supporting differences in risk behavior between delinquent and non-delinquent youth.

## Method

### Participants

Participants included 50 youth age 13–17 years (*M* = 16, SD = 0.97) from Eastern Illinois recruited for participation in a study comparing decision-making and behavioral outcomes between delinquent and non-delinquent youth. Approximately half of the sample (*n* = 23) was recruited for having engaged in antisocial behaviors, resulting in contact with the justice system such as theft, fighting or drug use. These youth, hereon referred to as ‘delinquent youth’, were recruited from one of three locations: an alternative school for students expelled or suspended for gross misconduct, the local juvenile detention center or the local parole and probation office. Although the original delinquent sample included *n *= 25 participants, two participants were excluded from the final sample due to excessive motion in the fMRI scanner. The other half of the sample (*n* = 27) was recruited from mainstream schools in the same geographic area. Participants were compensated US $50 for participation. Informed consent and assent were obtained in accordance with the university’s institutional review board.

Demographic information for the two groups is presented in Table 1. Non-delinquent and delinquent youth did not significantly differ in age or gender, but there were significantly more White participants in the non-delinquent group and significantly more Black participants in the delinquent group. Rates of self-reported risk-taking and externalizing symptoms between the two groups as well as rates of delinquency and institutional discipline experienced by youth in the delinquent sample are reported in Table 2. As expected, delinquent youth reported higher externalizing and risk behaviors than non-delinquent youth.

**Table 1.** Demographic information for non-delinquent and delinquent groups

	Group		Test
Demographic	Non-delinquent (*n *= 27)	Delinquent (*n *= 23)	(Non-delinquent–delinquent)
Age (years)	*M* = 15.85, SD = 0.64	*M* = 16.19, SD = 1.24	*t*(48) = −1.262, *P *= 0.213
Female	52% (*n *= 14)	52% (*n *= 12)	*t*(48) = −0.022, *P *= 0.982
White	78% (*n* = 21)	48% (*n *= 11)	*χ* ^2^(1) = 4.836, *P *= 0.028
Black	11% (*n *= 3)	52% (*n *= 12)	*χ^2^*(1) = 9.972, *P *= 0.002
Others	11% (*n *= 3)	0% (*n *= 0)	

**Table 2.** Self-reported externalizing behaviors and lifetime disciplinary history in delinquent and non-delinquent groups

Behavior	Group	Minimum	Maximum	Mean	SD	*t*(48)
Risk-taking	Non-delinquent	1	2.17	1.309	0.355	
	Delinquent	1.42	3.67	2.247	0.49	7.825^a^
Externalizing	Non-delinquent	1	2.69	1.456	0.427	
	Delinquent	1	4.38	2.551	1.114	4.726^a^
Number of suspensions^b^	Delinquent	0	8	4.708	2.985	
Number of expulsions^b^	Delinquent	0	3	0.792	0.833	
Number of arrests^b^	Delinquent	0	7	1.333	2.18	

a Means of non-delinquent and delinquent samples are significantly different at *P *< 0.001.

b Data not collected for non-delinquent youth.

### SART

**Fig. 1.**
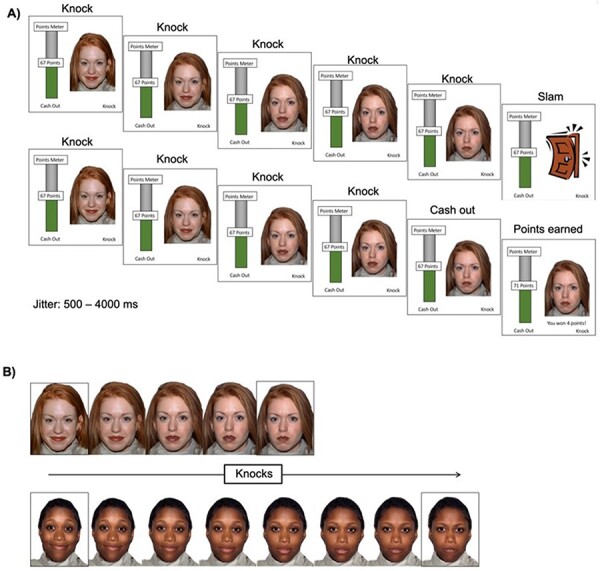
(A) Illustration of the ‘trick-or-treat’ task. Each decision was self-paced with a 500–4000 ms jitter between each event. If participants knocked the door until the resident’s face reached 50% anger, the door would slam (top half of figure). Participants could also choose to cash in any time (bottom half of figure). (B). Illustration of variability in slam points. In the top half of the figure, the resident’s expression changes quickly, resulting in a slam after only four knocks. In the bottom half of the figure, the resident’s expression changes slower, resulting in a slam after seven knocks.

Participants completed the SART (adapted from Humphreys *et al.*, 2016) based on the well-validated BART (Lejuez *et al.*, 2002). The SART was developed to index risk-taking as a function of sensitivity to social feedback. Participants were told that they would be playing a ‘trick-or-treat’ game in which they would approach 24 people at their house (Figure 1). Each house corresponded with one trial. At each house, participants could knock on the door by making a button press (accompanied by a knocking sound) to earn points. The screen displayed the resident’s face and always started with a 100% happy expression. With each knock, the face morphed from happy to neutral to angry, at which point the door would slam (accompanied by a loud slamming sound) and participants would lose all points earned in that trial. The slam point, or number of knocks leading to a door slam, varied between 3 and 10 knocks (*M = *6.5, SD = 1.29). There were three trials per slam point (e.g. 3 out of 24 houses had a slam point of 10). Participants could ‘cash in’ (accompanied by a rising note sound) the points earned on each door at any point before the door slam and move on to the next trial. A running total of points were presented as a points meter on the left side of the screen.

Participants saw 12 individual faces (4 White, 4 Black and 4 Asian; all faces were female) twice each during the task. Each unique face appeared at least once before any face reappeared. The task was self-paced and did not advance unless participants decided to either knock or cash in. A random jitter (500–4000 ms) followed each event (i.e. (i) a knock, (ii) a new face after the decision to cash in and (iii) a new face following a slam). Faces were presented in a fixed order. Prior to the scan session, research assistants trained participants on the task. To incentivize performance, participants were shown a box of age-appropriate prizes (e.g. snacks and movies) and were told that they could select prizes based on the points earned during the game. In reality, all participants were allowed to choose three prizes.

Whereas the BART offers no information about the balloon’s explosion point, the SART offers dynamic feedback (i.e. morphing face) about each door’s slam point, allowing participants to modify their behavior based on the anger level displayed in the face. This unique feature of the SART allowed us to measure participants’ sensitivity to risk. Behaviorally, we examined how knock decisions (i.e. total number of knocks on each trial) varied as a function of risk (i.e. the trial-specific slam point). At the neural level, we had two conditions of interest: (i) neural sensitivity to risk (i.e. when deciding to knock, does brain activation change as the face gets angrier?) and (II) neural sensitivity to reward (i.e. when deciding to cash in, does brain activation change as reward value increases?).

### fMRI data acquisition and analysis

A 3 Tesla Siemens Trio MRI scanner was used to collect imaging data. Structural scans consisted of a T1* magnetization-prepared rapid-acquisition gradient echo (MPRAGE; Repitition Time (TR) = 1.9 s; Time-to-Echo (TE) = 2.3 ms; Field-of-View (FOV) = 230; matrix = 256 × 256; sagittal plane; slice thickness = 1 mm; 192 slices) and a T2*-weighted, matched-bandwidth (MBW), high-resolution anatomical scan (TR = 4 s; TE = 64 ms; FOV = 230; matrix = 192 × 192; slice thickness = 3 mm; 38 slices). During the trick-or-treat task, T2*-weighted echoplanar images (EPI; slice thickness = 3 mm; 38 slices; TR = 2 s; TE = 25 ms; matrix = 92 × 92; FOV = 230 mm; voxel size 2.5 × 2.5 × 3 mm^3^) were acquired. MBW and EPI scans were obtained using an oblique axial orientation in order to maximize brain coverage.

The Statistical Parametric Mapping (SPM8; Wellcome Department of Cognitive Neurology, Institute of Neurology, London, UK) statistical package was used for data preprocessing and analysis. Preprocessing involved correcting for head motion using spatial realignment. We censored TRs with 2 mm or more of absolute motion in any direction. There were no significant group differences in movement between the delinquent and non-delinquent groups (*t*(48) = 0.974, *P *= 0.335). Next, all images were coregistered to the high-resolution T1* MPRAGE structural scan and segmented into gray matter, white matter and cerebrospinal fluid. MWB and EPI images were warped into the standard stereotactic space defined by the Montreal Neurological Institute (MNI) and the International Consortium for Brian Mapping by applying the transformation matrices used in MPRAGE segmentation. To increase the signal-to-noise ratio in the functional images, EPI images were smoothed using an 8 mm Gaussian kernel, full-width-at-half maximum. Each trial was then convolved with a canonical hemodynamic response function using the general linear model in SPM8. Finally, a high-pass temporal filter (cutoff = 128 s) was applied to the time series in order to remove low-frequency drift and a restricted maximum likelihood algorithm with an autoregressive model order of 1 was used to estimate serial autocorrelations.

The SART is an event-related design. We included general linear models for each condition of interest (i.e. knock decisions, cash-in decisions and slam events) in the fixed effect models. Knock decisions on doors ending in cash-ins were modeled separately from knock decisions on doors ending in slams. As in previous research (Lejuez *et al.*, 2002; Telzer *et al.*, 2015), analyses only included knock decisions made during doors ending in cash-ins because slams artificially curtail participants’ knocking behavior. Each trial was modeled from the onset of the event to the point at which participants made their decision. The jittered intertrial periods between knock and cash-in decisions were not modeled and served as an implicit baseline.

To examine how neural activation changed as a function of risks (i.e. increasing knocks) and rewards (i.e. increasing receipt of points), a parametric modulator (PM) was included for our two conditions of interest: knock decisions and cash-in decisions. For knock decisions, the PM values represented the cumulative number of knocks within a trial (i.e. house). The PM values for each knock were centered within person around the average number of cumulative knocks within the trial. This PM allowed us to examine how the brain tracked increasing risk. For cash-in decisions, PM values represented the total number of knocks for each trial (i.e. the total risk that was taken for each house). The PM values for each cash-in trial were centered within person around the average number of knocks for the entire task. This PM allowed us to examine how the brain tracked increasing reward receipt, as more knocks equated to more points earned. This analytic approach was used to be consistent with prior studies using the SART task (e.g. McCormick *et al.*, 2018; Van Hoorn *et al.*, 2018).

Random-effects group-level analyses included the individual-level contrast images. Group-level analyses were conducted using GLMFlex, which removes outliers and sudden activation changes, partitions error terms, analyzes all voxels containing data and corrects for variance–covariance inequality (http://mrtools.mgh.harvard.edu/index.php/GLM_Flex). Our primary analyses included two-sample *t*-tests comparing the delinquent to non-delinquent groups on our contrasts of interest. Because slam events occurred too infrequently, this condition was not included in the analyses. We corrected all analyses for multiple comparisons using Monte Carlo simulations through 3DClustSim (updated version November 2016) in the software package AFNI (Ward, 2000) and accounted for the intrinsic smoothness of the data with the -acf function within the 3dFWHMx command. We used a voxel-wise threshold of *P *<0.005, corresponding to *P* <0.05, family-wise error cluster-corrected. All reported results are available on NeuroVault (Gorgolewski *et al.*, 2015; see https://neurovault.org/collections/12312/).

## Results

We conducted all behavioral analyses using Mplus v.8. To compare general performance on the SART between delinquent and non-delinquent youth, we first conducted independent samples *t-*tests for the total number of knocks (i.e. overall risk-taking). No significant differences in risk-taking (i.e. knocking) emerged between delinquent and non-delinquent youth, *t*(48) = 1.797, *d *= 0.51, *P = *0.079.

To examine the behavioral sensitivity to risk, we estimated a model with risk-taking—indexed as the number of knocks for each trial—as the dependent variable, trial-specific slam point as a within-person independent variable (higher values indicate the face gets angry slower) and group (delinquent = 1) as a between-person independent variable. Including trial-specific slam point in the model allowed us to explore how risk behavior changed as a function of anger rate. Additionally, we examined the cross-level slam point × group interaction to explore whether sensitivity to risk differed between delinquent and non-delinquent youth. To control for learning effects (e.g. Van Hoorn *et al.*, 2018), we entered trial number, previous trial outcome (1 = slam) and current trial outcome (1 = slam) as within-person covariates in the model.

**Fig. 2.**
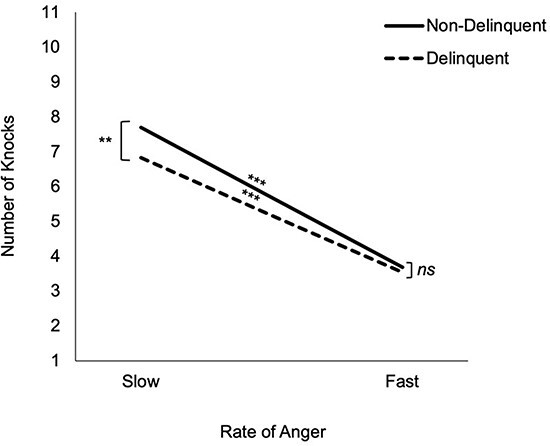
Illustration of the cross-level interaction between the slam point and the group. The rate of anger represents the various slam points across trials (slow = 10 knocks; fast = 3 knocks). On slow trials, non-delinquent youth knocked more frequently than delinquent youth. There were no group differences in knocks on fast trials.

Results from the multilevel model indicated that as slam point increased, risk-taking increased (Table 3). In other words, on trials with a slower transition to an angry face, participants knocked more frequently than on trials with a faster transition to an angry face. Furthermore, group moderated the effect of slam point on knock decisions. To explore this interaction, we examined the association between the slam point and the number of knocks in the delinquent and non-delinquent groups separately. Results indicated that both delinquent (*B* = 0.503, SE = 0.028, *β *= 0.655, *P *< 0.001) and non-delinquent (*B* = 0.588, SE = 0.024, *β *= 0.726, *P *< 0.001) youth knocked less frequently as slam point decreased (i.e. faces became angry faster). To further unpack the interaction, we explored group differences in risk-taking between trials with slow *vs* fast slam points. To do this, we tested for group differences in the number of knocks when the slam point was fastest (3 knocks to slam; face gets angry quickly) and when the slam point was slowest (10 knocks to slam; face gets angry slowly). Results indicated that delinquent youth evinced less risk-taking (i.e. cashed out sooner) than non-delinquent youth on slow trials (*B* = −0.959, SE = 0.362, *β *= −0.211, *P *= 0.008), but that there were no differences in risk-taking on fast trials (B = −0.094, SE = 0.066, *β *= −0.115, ns). Thus, whereas delinquent and non-delinquent youth made comparably risky decisions on trials with a fast slam point, they were less risky on trials with a slow slam point (Figure 2).

**Table 3.** Results from the multilevel model examining differences in sensitivity to social feedback between delinquent and non-delinquent youth on social risk-taking task

Outcome: number of knocks		*B*	SE (*B*)	*β*
Within	Trial	0.013	0.009	0.055
	Previous outcome	−0.452^***^	0.112	−0.079
	Current outcome	0.27*	0.124	0.046
	Slam point	0.525^***^	0.032	0.752
Between	Group	−0.506*	0.248	−0.294
Cross-level	Slam point × group	−0.101*	0.042	^a^

*Note.* Trial number, previous outcome (1 = Slam), and current outcome (1 = Slam) were included as covariates to control for learning effects on decision-making. The slam point represents how quickly the face morphed into an angry expression; higher values indicate that participants could knock more before reaching the slam point. Group represents the delinquent (1) and non-delinquent (0) youth. Coefficients for the main effects are from a model excluding the interaction term. ^***^P < 0.001; * *P *< 0.05; ^a^ Standardized coefficients are not available for cross-level interactions in Mplus.

### Neural sensitivity to risk and reward

At the whole-brain level, we examined neural tracking of risks and rewards by measuring changes in neural activation as a function of changes in risk (i.e. angry faces) and rewards (i.e. points earned). We started by exploring the main effects of risk and reward within the full sample (both delinquent and non-delinquent youth). In the first analysis, we modeled neural tracking of increasing risk by examining activation during knock decisions with the cumulative number of knocks within trials included as the PM. Results indicated increased neural tracking of risk (i.e. brain activation increased as faces became angrier) in regions such as the VS and insula. Additionally, we observed decreased neural tracking of risk (i.e. brain activation decreased as faces became angrier) in regions such as the fusiform gyrus, orbitofrontal cortex and dorsolateral prefrontal cortex (see Table 4 for the full list of regions). Next, we modeled neural tracking of increasing reward by examining activation during cash-in decisions with the total number of knocks within trials included as the PM. Results showed increased neural tracking of reward (i.e. brain activation increased as earnings increased) in the VS, insula, fusiform gyrus and dorsolateral prefrontal cortex (see Table 5 for the full list of regions).

**Table 4.** Regions showing increased and decreased neural tracking of risk (angry faces) during knock decisions

				MNI coordinates		
	Region	*k* ^a^	*t*-value	*x*	*y*	*z*
Increased tracking	Right anterior insula	368	6.960	36	23	10
	Right ventral striatum	183	5.333	12	8	−5
	dorsal anterior cingulate cortex	502	4.989	9	26	34
	Left anterior insula	272	5.506	−30	20	−8
	Right precentral gyrus	280	5.134	39	−13	64
Decreased tracking	Right fusiform gyrus	32884^a^	7.411	45	−64	−17
	Left fusiform gyrus	^a^	6.436	−45	−58	−17
	Orbitofrontal cortex/ventromedial prefrontal cortex	^a^	7.264	−9	62	−5
	Dorsolateral prefrontal cortex	266	5.824	48	47	7

*Note.* The map was thresholded at *P *< 0.005. Monte Carlo Simulation yielded a minimum cluster size of 139 for whole-brain analysis. ^a^ Regions part of the same cluster share a superscript.

**Table 5.** Regions showing increased neural tracking of rewards during cash-in decisions

			MNI coordinates		
Region	*k*	*t*-value	*x*	*y*	*z*
Dorsal anterior cingulate cortex	493	4.358	−6	35	7
Left anterior insula	182	4.301	−36	14	−2
Left putamen/caudate	144	3.93	−21	−4	19
Right dorsolateral prefrontal cortex	471	4.554	36	35	37
Left dorsolateral prefrontal cortex	615	4.348	−48	26	28
Right fusiform gyrus	2465	3.974	42	−55	−11
Left fusiform gyrus	624	3.77	−45	−55	−14

*Note.* The map was thresholded at *P *< 0.005. Monte Carlo Simulation yielded a minimum cluster size of 135 for whole-brain analysis.

**Fig. 3.**
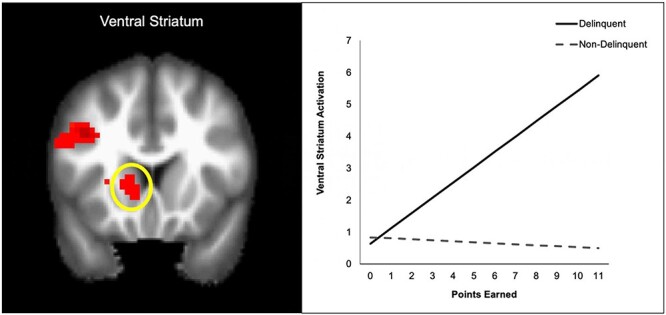
Differences in neural tracking of reward (cash-ins) between non-delinquent and delinquent youth in the VS.

Finally, we examined group differences in neural tracking of risk and reward by conducting an independent sample *t-*test on the analyses for knock decisions and cash-ins (including number of knocks as the PM). Results indicated no significant group differences in neural tracking of risk. However, we found group differences in neural tracking of reward (i.e. cash-ins). Compared to non-delinquent youth, delinquent youth evinced greater neural tracking of rewards (i.e. brain activation increased as earnings increased) in regions such as the VS, inferior parietal lobule (IPL) and fusiform gyrus (Table 6 for the full list of regions). For descriptive purposes, we extracted parameter estimates of signal intensity from the VS and plotted activation across levels of reward for the delinquent and non-delinquent groups separately. As shown in Figure 3, delinquent youth evinced increasing sensitivity to obtaining higher rewards (i.e. points earned) in the VS, whereas non-delinquent youth showed relatively low VS activation across reward value. Due to an insufficient number of trials, we did not have enough statistical power to parallel the behavioral results and examine the neural findings separately for ‘fast’ and ‘slow’ trials.

**Table 6.** Regions showing greater neural tracking during reward receipt (cash-ins) in delinquent than non-delinquent youth

			MNI coordinates		
Region	*k*	*t*-value	*x*	*y*	*z*
Left VS	472	3.146	−12	17	−2
Left IFG	576	3.980	−33	38	7
Right IPL	301	3.855	39	−52	43
Right fusiform gyrus	284	3.005	30	−61	−5
Left fusiform gyrus	300	2.691	−30	−70	−11

*Note.* The map was thresholded at *P *< 0.005. Monte Carlo Simulation yielded a minimum cluster size of 140 for whole-brain analysis.

## Discussion

The present study compared risk behavior and neural sensitivity to risks and rewards between delinquent and non-delinquent youth. Using an experimental socioemotional risk task that provided real-time feedback on the amount of risk incurred, we explored dynamic changes in brain activity during decision-making. Whereas risk-taking was similar between delinquent and non-delinquent youth on high-risk trials with a fast slam point (i.e. fewer opportunities to earn rewards), delinquent youth were less risky on trials with a slow slam point (i.e. more opportunities to earn rewards). At the neural level, delinquent youth demonstrated greater VS tracking of rewards than non-delinquent youth. Together, these findings suggest that delinquent youth do not ubiquitously take more risks than their non-delinquent peers. Furthermore, delinquent youth demonstrate heightened neural sensitivity to rewards than non-delinquent youth.

Since prior work suggests that delinquent youth are less risk-averse (Frick *et al.*, 2003) and more sensitive to rewards (Hawes *et al.*, 2021) than non-delinquent youth, we expected that delinquent youth would take advantage of the opportunity to acquire as many rewards as possible and therefore take more risks than non-delinquent youth. This hypothesis was not supported. Instead, delinquent youth took the same amount of risks as non-delinquent youth on trials with a fast slam point and took fewer risks on trials with a slow slam point. Differences in delinquent youth’s risk behavior between fast and slow trials demonstrate that delinquent youth integrated feedback into their decision-making and used that information to cash-in sooner.

There are two potential explanations for less risk-taking among delinquent youth on the slow trials: either delinquent youth were more sensitive to risk (i.e. heightened sensitivity to angry faces, which provided information about when the door was going to slam), or they were more sensitive to reward (i.e. they made a conscious decision to cash-in earlier to obtain an immediate reward). Indeed, some research suggests that delinquent youth are more averse to negative socioemotional stimuli than non-delinquent youth (Eftekhari *et al.*, 2004) and avoid situations that induce negative feelings (Richter *et al.*, 2002). However, other work suggests that delinquent youth have an overall blunted response to socioemotional cues (Gonzalez-Gadea *et al.*, 2014). We speculate that if delinquent youth were highly sensitive to the angry faces, they would have been more risk-averse on the fast trials, when the faces became angry sooner, rather than on slow trials. For this reason, we suspect that delinquent youth cashed in earlier with the intention of obtaining more immediate rewards, consistent with prior work suggesting delinquent youth struggle to delay gratification (Matthys *et al.*, 2013). Nevertheless, future research will need to replicate these findings. One important consideration is the confounder between risk (impending door slam) and the emotional stimulus of an angry face. Thus, future work should compare delinquent youth’s sensitivity to risks and rewards on tasks with and without socioemotional feedback.

Results from the fMRI analyses indicated that delinquent youth evinced linear increases in VS activation to rewards when cashing in their points. These results suggest that delinquent youth may have been more sensitive than non-delinquent youth to rewards, consistent with prior research (Hawes *et al.*, 2021). The VS is involved in tracking the subjective value of stimuli and processing the presence or expectation of rewards (Knutson *et al.*, 2001; Eldar *et al.*, 2016). Prior work has implicated VS activity in antisocial behavior (Holz *et al.*, 2017). For example, heightened VS activity during risk-taking predicts earlier binge drinking among youth, over and above behavioral assessments of risk (Morales *et al.*, 2018). Hyperactivity of the VS is also linked to preferences for smaller, immediate rewards over larger, delayed rewards (Hariri *et al.*, 2006). These findings offer useful contextual information about the potential neuropsychological processes associated with decision-making. Thus, our findings present an alternative to theories purporting that delinquent behavior is due to dampened risk or threat processing (Du, 2019) and suggest that activity in reward-processing brain regions like the VS may be a key neurocognitive mechanism contributing to increased risk behavior among delinquent youth.

In addition to the VS, delinquent youth showed heightened neural tracking of rewards during cash-in decisions in the fusiform gyrus, inferior frontal gyrus (IFG) and IPL. The fusiform gyrus is largely involved in facial (Iidaka, 2013) and emotional information processing (Frank *et al.*, 2019; Graham *et al.*, 2021). Thus, greater neural tracking of reward in this region among delinquent youth may suggest greater encoding of angry faces as the reward value increased. Relatedly, the IFG is involved in monitoring and cognitive control (Dosenbach *et al.*, 2008) as well as emotional processing (Rosen *et al.*, 2017). Coupled with greater activation in the IPL, which has been previously linked to executive control (Dosenbach *et al.*, 2008) and monitoring feedback (Vickery and Jiang, 2009), greater neural tracking of reward in these regions may indicate regulation of affective responses to angry faces, perhaps in order to cash in. These findings show that delinquent youth are not insensitive to risk or to socioemotional feedback but do indeed integrate this information into their decision-making. In future research, it will be important to directly test whether delinquent youth process information about risk differently when that information is social versus non-social.

Somewhat surprisingly, delinquent and non-delinquent youth showed no significant group differences in neural tracking of risks (i.e. faces becoming angrier as participants got closer to losing all their points). This finding is in line with theories suggesting that delinquent youth are not averse to social threat (Frick *et al.*, 2003). However, this is not to say that delinquent youth completely disregarded social information on the task, as indicated by the observed activation in the fusiform gyrus during cash-in decisions. Furthermore, it is important to remember that the behavioral results suggested differences in risk-taking between delinquent and non-delinquent youth on ‘slow’ trials only. Therefore, it is possible that group differences in neural tracking of risk would have emerged had we been able to compare the neural findings between ‘slow’ and ‘fast’ trials.

The findings from this study offer an important contribution to the neurodevelopmental literature, which currently lacks a clear understanding of how risky decision-making processes differ between youth engaging in delinquent *vs* normative patterns of risk behavior. Nevertheless, there are a couple of limitations in this study worth considering when interpreting the results. In addition to the small sample size, it is important to acknowledge the over-representation of Black adolescents in our sample of delinquent youth. Black adolescents’ experiences with authority figures are subjectively and quantitatively different from their White peers. With a larger sample size, future work may be able to disentangle whether there are racial and ethnic differences in neural processing of risk and reward and be able to frame those differences in key contextual factors such as experiences with discrimination. Additionally, socioeconomic data were not available for the subjects. Prior work has shown that socioeconomic factors like lower parent education (Brieant *et al.*, 2020) and worse neighborhood quality (Gonzalez *et al.*, 2016) are associated with greater risk-taking and heightened neural sensitivity in mesolimbic reward areas during adolescence. Thus, future work should consider how socioeconomic factors interact with adolescents’ sensitivity to risks and rewards and their subsequent decision-making.

Furthermore, the behavioral analyses in this study do not directly parallel the neural analysis. Specifically, the behavioral data suggested that risk behavior differed between slow and fast trials. However, we were underpowered at the neural level to break down our analyses into slow and fast trials. Thus, although we did not find group differences in neural tracking of risk (i.e. changing emotional expressions) overall, it is possible that group differences would have emerged when splitting the data into fast and slow trials. In spite of this limitation, the neural findings in this study offer unique information about the neuropsychological processes associated with risk behavior between delinquent and non-delinquent youth that is currently limited in the neurodevelopmental literature.

The present study increases our understanding of the neuropsychological pathways that may distinguish youth who engage in normative versus non-normative and persistent patterns of risk behavior. Differentiating increases in normal tolerance of risk from increased propensity to commit delinquent acts is paramount for improving adolescent outcomes and requires assessment of both behavioral preferences and neural responses to risk and reward systems. By examining these processes with an ecologically valid paradigm, we found that delinquent youth did not show greater preference for risk nor did they show reduced neural tracking of risk than their non-delinquent counterparts, but did show greater neural tracking of reward. This suggests that reward processing may be one key neural process associated with delinquent youths’ decision-making in risky contexts. Examining how these neuropsychological mechanisms interact in delinquent behavior could help make treatments more efficacious and improve our ability to minimize delinquency among youth.

## Data Availability

The data underlying this article will be shared on reasonable request to the corresponding author.
